# Fitness Regulates Non‐Native Species Recovery in a Simulated Trojan M_YY_ Brook Trout Application

**DOI:** 10.1111/eva.70335

**Published:** 2026-10-14

**Authors:** K. Davenport, D. Alò, C. C. Day, R. K. Simmons, E. L. Landguth, A. R. Whiteley

**Affiliations:** ^1^ Wildlife Biology Program, Franke College of Forestry and Conservation University of Montana Missoula Montana USA; ^2^ Computational Ecology Lab University of Montana Missoula Montana USA; ^3^ Seattle City Light Seattle Washington USA

**Keywords:** agent‐based modeling, CDMetaPOP, hybrid index, negative heterosis, supermales, YY‐male

## Abstract

Trojan Y chromosome methodologies have been developed for bio‐control of non‐native freshwater fish. Individuals with an altered sex chromosome genotype are introduced into the environment to mate with wild conspecific and change the population sex ratios, with the goal of eradicating local populations. This strategy preserves native stocks and can be effective when other methods are impracticable. Applications employing Trojan males with two Y chromosomes (M_YY_) to control non‐natives have shown promise for eradication at small spatial scales when used with manual suppression. Failure to eradicate may have consequences for the fitness and resiliency of the recovering population and remains untested. Population bottlenecks due to suppression and M_YY_ introductions may cause inbreeding depression while admixture with hatchery M_YY_ may counteract inbreeding depression and result in outbreeding depression. We used a spatial demogenetic and individual‐based simulation model to examine the fitness consequences of inbreeding and outbreeding depression on the recovery of a non‐native Brook Trout ( *Salvelinus fontinalis* ) population targeted for removal in northeastern Washington State, USA. Simulations predicted genetic and fitness consequences, as well as the extent and timing of population recovery following suppression and introduction of M_YY_. We found that inbreeding depression rapidly decreased overall population size and outbreeding depression was important for counteracting the fitness advantage of admixture from genetically divergent M_YY_. Additionally, combined effects of inbreeding and outbreeding depression prevented the population from recovering to pre‐suppression abundance compared to each in isolation. This represents the first study demonstrating the demogenetic and fitness consequences of inbreeding and outbreeding depression from a M_YY_ program when non‐native species eradication is not achieved. Results suggest that the combination of negative fitness effects may limit the ability of the surviving non‐native population to recover and reestablish once treatments cease, indicating that such treatments may be beneficial even if eradication is not ultimately achieved.

## 1 Introduction

Non‐native freshwater species are a significant threat to biodiversity, causing severe challenges for the conservation and management of native species (Crystal‐Ornelas and Lockwood 2020; Jelks et al. 2008). The use of M_YY_ treatments (Trojan Y‐Chromosome, TYC) is one method to control non‐native fish species where chemical treatment is either not possible (Britton et al. 2011) or their mechanical removal alone is unlikely to remove all individuals in a population (Meyer et al. 2006). The M_YY_ treatments involve introducing hatchery‐produced males of the same species with two Y chromosomes to shift the sex ratio of future generations toward male until the population is eradicated (Cotton and Wedekind 2007; Gutierrez and Teem 2006; Schill et al. 2016). M_YY_ treatments are often used in conjunction with suppression to accelerate the rate of the decline of the non‐native population compared to suppression alone (Day et al. 2021; Gutierrez and Teem 2006; Kennedy et al. 2018; Schill et al. 2017). Modeling and on‐the‐ground M_YY_ programs focused on non‐native Brook Trout populations in the Western US have shown promise. However, factors such as survival, reproductive fitness, and dispersal rate of M_YY_ may impact the overall success of these programs (Day et al. 2021, 2020; Miller et al. 2024, 2026; Schill et al. 2017; Teem et al. 2014).

Although extirpation of non‐native populations through shifting the sex ratio is the primary goal of programs combining suppression and M_YY_ treatments, the method may also force the population through a genetic bottleneck (Allendorf et al. 2012). Bottlenecks increase the chance of inbreeding among related individuals and can generate harmful effects through increasing homozygosity. This phenomenon, known as “inbreeding *depression*” (ID), reduces fitness by affecting vigor and reproductive rate, potentially increasing the rate of population decline (Emlen 1991; Falconer and MacKay 1996; Kardos et al. 2015). Admixture between hatchery M_YY_ and wild fish might also cause “outbreeding *depression*” (OD; or negative heterosis) by admixing genetically divergent individuals (Lynch 1991), which can decrease fitness through genomic incompatibilities or maladaptation with gene variants less fit for their environment (Christie et al. 2014; Gharrett et al. 1999; McGinnity et al. 2003; Miller et al. 2004). Alternatively, adding M_YY_ may also introduce a sustained pulse of admixture from genetically divergent sources leading to heterosis (or *hybrid vigor*), where admixed crosses have higher fitness than their parents, dampening the negative effects of ID and/or OD. However, assuming a wild population is adapted to its environment, it is expected that any genetic change via admixture would result in a reduction of fitness, also identified as genetic load (Bertorelle et al. 2022). Therefore, if eradication is not achieved via M_YY_ treatments, the genetic load generated by inbreeding and outbreeding could continue to limit the ability of the non‐native population to recover and re‐establish through lower fitness of offspring.

The outcome of this complex mixture of induced inbreeding along with outbreeding from a domesticated source population is challenging to predict. Although simulation modeling has been used to evaluate the factors impacting successful eradication using M_YY_ treatments, such as the size of M_YY_ released or the annual stocking rate (Schill et al. 2017; Day et al. 2021), the demographic and genetic consequences of not achieving eradication remain untested. Eradication efforts are likely to increase the genetic load of the remnant non‐native population in cases where the population is not eradicated, since previously masked mutations can become expressed due to the fixation of deleterious mutations after prolonged bottlenecks (Bertorelle et al. 2022; Dussex et al. 2023).

In this study, we explored the effects of selection on the rate and magnitude of recolonization. We simulated demographic and genetic dynamics of a non‐native population after suppression and M_YY_ introduction (hereafter treatments) have ceased and complete eradication failed to occur. Building on the framework used by Day et al. (2020, 2021), we modeled treatments using Brook Trout ( *Salvelinus fontinalis* ) into an established population of non‐native wild Brook Trout using individual‐based simulations that included spatial and genetic components. Non‐native Brook Trout are a well‐suited model organism that has previously been studied using simulations that assessed how management factors affect the success of M_YY_ treatments. We examined how the isolated and combined fitness effects of inbreeding and outbreeding influence overall population size, sex ratio, homozygosity, and mortality due to selection once treatments have ceased. Under a baseline scenario with no selection, we predict that populations will be genetically bottlenecked but make a full demographic recovery. When inbreeding and/or outbreeding occur, we predict that the bottleneck will result in reduced population fitness, thereby increasing recovery time and reducing the recovered population size. When only inbreeding is implemented, we predict a reduced effect of the homozygosity fitness penalty due to outbreeding with hatchery males.

## 2 Methods

### 2.1 Study Site

The spatial extent of this study is the occupied range of Brook Trout in the Sullivan Creek Watershed in Washington State, USA (Figure 1). Sullivan Creek (roughly 32 km of occupied habitat) is a large tributary to the Lower Pend Oreille River. Non‐native Brook Trout were introduced as early as 1913, with multiple subsequent stocking events occurring throughout the century (Johnson 1913), and they now occur throughout most of the mainstem and lower portions of tributaries of Sullivan Creek. Suppression efforts for Brook Trout began in 2016, and M_YY_ introductions since 2018, creating opportunities to study M_YY_ Brook Trout using field studies and simulations (Day et al. 2020, 2021).

**FIGURE 1:**
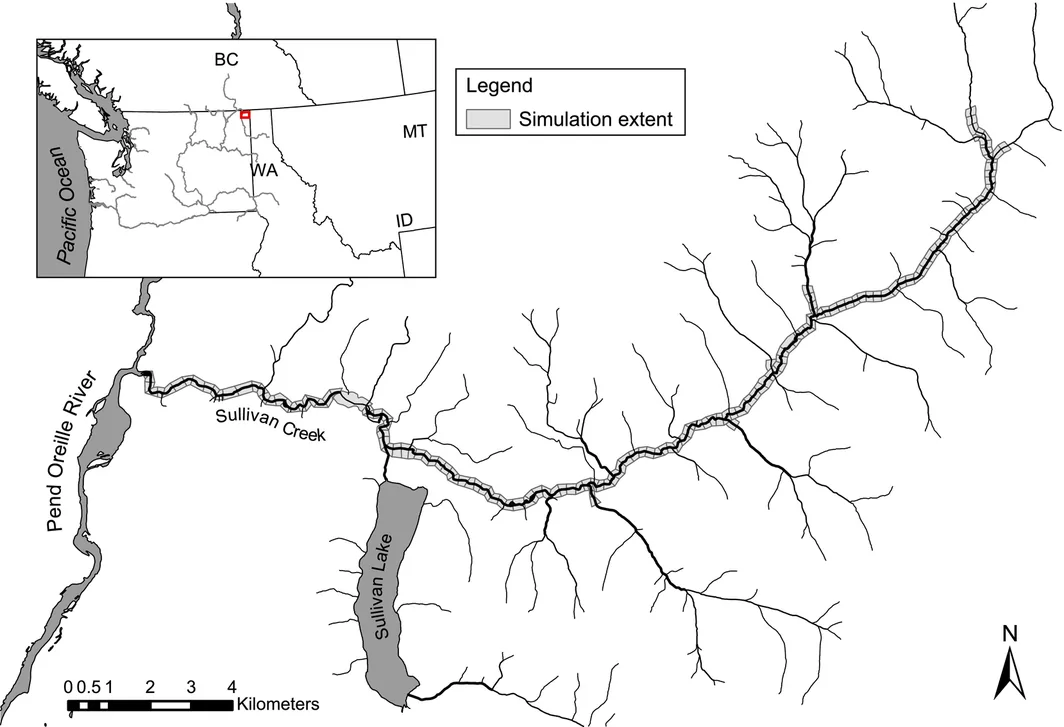
Study area. Location of the Sullivan Creek watershed, tributary of the Pend Oreille River System in northeastern Washington State, USA, and the simulation extent (gray buffering).

### 2.2 Model Description

The simulation of genetics, population dynamics, movement, suppression, and introduction of M_YY_ was completed using CDMetaPOP version 2.51 (Day et al. 2023; Landguth et al. 2017). CDMetaPOP is a demogenetic model that simulates the movement and exchange of genetic material of individuals that occupy discrete patches (subpopulations) arranged across a heterogeneous landscape (Landguth et al. 2017). Within the model, each time step represents 1 year and allows individuals to grow (as a function of temperature), mature, mate, and reproduce (as a function of size), and move (as a function of riverine distance). Additionally, size‐structured density‐dependent mortality occurs annually. Previous studies incorporated (into CDMetaPOP) mechanical suppression through electrofishing (Day et al. 2018) and M_YY_ introduction (Day et al. 2020). Our study implements these earlier iterations (see Table S1) and additionally incorporates the fitness effects of ID and OD.

### 2.3 Introduction of M_YY_

To simulate the introduction of M_YY_, we used the previously developed module within CDMetaPOP that allows for the introduction of fish at designated time steps. Fish can be assigned sex chromosomes (XX, XY, and YY), and individuals with YY chromosomes were introduced into the upper portion of the system consistent with the current stocking of M_YY_ in Sullivan Creek. Introduced M_YY_ were assigned their own allele frequencies (as described below) and an independent set of parameters for mortality, maturation, fecundity, and movement based on Day et al. (2020; Table S1).

### 2.4 Allele Frequency Parameterization

Patch genetic data for the wild Brook Trout populations were initiated with allele frequencies generated from empirical adult Brook Trout genotypes using a 240 loci SNP panel (Davenport 2023) from seven locations within the Sullivan Creek system. Locations were selected if at least 20 individuals had been sampled with genetic data. Allele frequencies for M_YY_ Brook Trout were generated using genotypes (using the same SNP panel) from the Washington Department of Fish and Wildlife Molecular Genetics Lab for samples from Hayspur Hatchery in Idaho (*N* = 386), which provides the M_YY_ Brook Trout currently being introduced into the Sullivan Creek system. Loci that had missing data for at least 10 individuals per population were removed, leaving 219 loci. All loci were unlinked and sexes were pooled since no differences were found between male and female allele frequencies.

Since estimates of individual inbreeding coefficients are sensitive to number of loci (Kardos et al. 2015; Nietlisbach et al. 2017), the original SNPs dataset was duplicated to simulate a larger sample size. *R* ^2^ was calculated for the correlation between pedigree and observed homozygosity (hom_o_) for 500, 1000, 5000, and 10,000 loci. Loci were replicated to 5000 until the *R* ^2^ (0.918) was not sensitive to further increases, creating a good balance between run time and *R* ^2^ value.

### 2.5 Study Design

#### 2.5.1 Patch Parameterization

The study area was divided into 152 discrete patches (each representing an approximately 200‐m stream reach) within the area of Brook Trout distribution in Sullivan Creek. Carrying capacity, habitat suitability, temperature, capture probability, and resistance to movement between patches were parameterized at the patch level following Day et al. (2020; Table S1). To stabilize population dynamics, genetic exchange was initiated 10 years into the simulations with wild Brook Trout allele frequencies (see Allele Frequency Parameterization) specific for each location. Each scenario was replicated 10 times and ran for 70 years.

#### 2.5.2 Null Scenario

The null scenario included no treatments and no fitness penalties to provide a demographic and genetic frame of reference for the alternative scenarios described next.

#### 2.5.3 Alternative Fitness Scenarios

To demonstrate the effects of ID and/or OD on the recovery of the wild Brook Trout population after treatments ceased, we executed several alternative scenarios: (a) treatments + no fitness penalty (baseline), (b) treatments + ID fitness penalty, (c) treatments + OD fitness penalty, and (d) treatments + ID + OD fitness penalties combined (Figure 2a).

**FIGURE 2:**
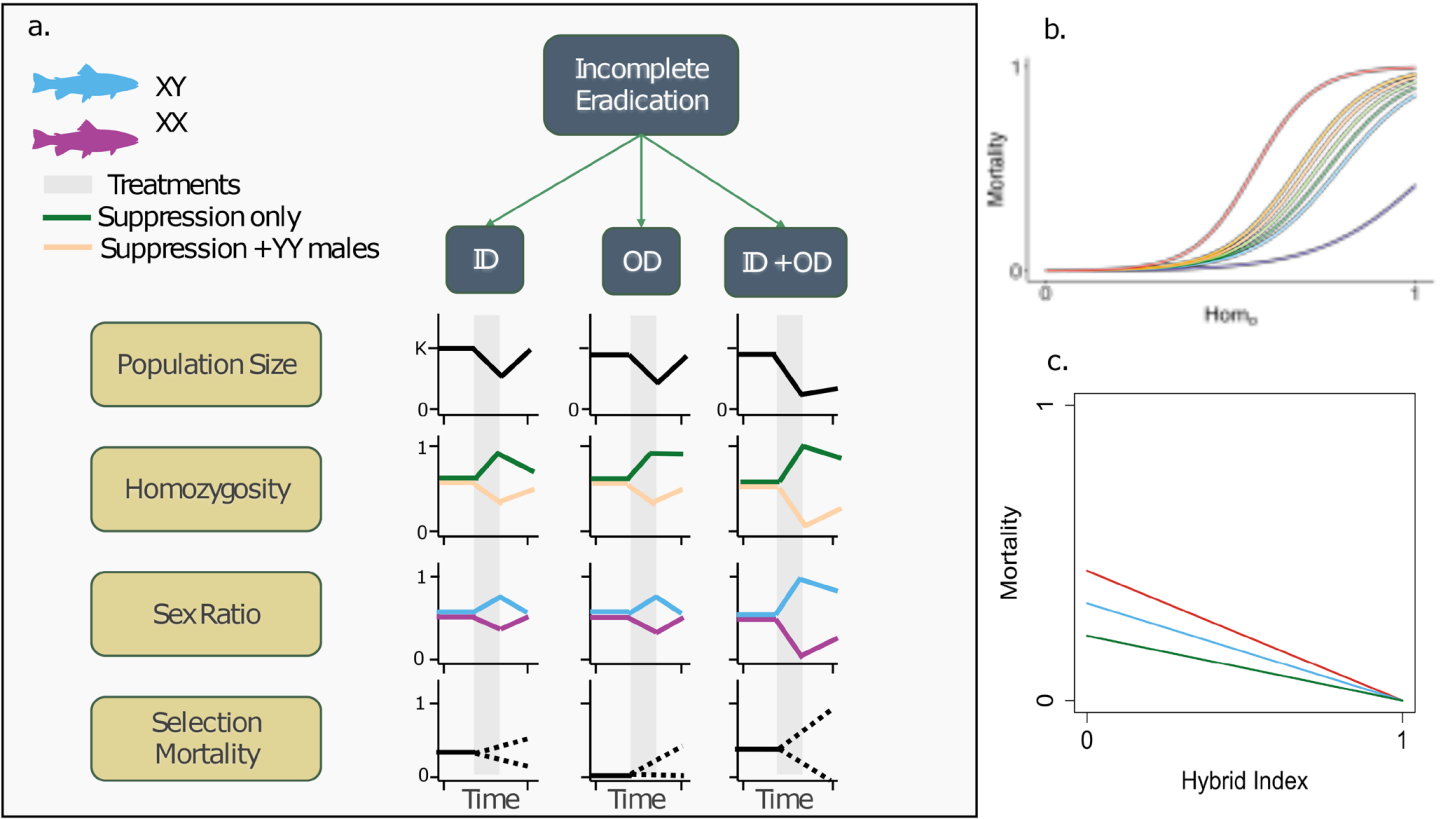
Conceptualized hypotheses. (a) Simplified theoretical expectations of incomplete eradication outcomes after treatments (grey vertical timeframe bar) with suppression only (green line) or suppression and M_YY_ introductions (cream line). Homozygosity dynamics carry different and contrasting implications for suppression alone (electrofishing) versus suppression plus M_YY_ introductions and these expectations are represented with different colors (green vs. cream lines). For these predictions, we consider effects of selection against homozygotes (ID), admixture from M_YY_, and selection due to negative heterosis (OD). (b) Logistic regression describing the alternative selection penalties as a function of homozygosity (ID). (c) Linear regression describing alternative selection penalties as a function of OD, which is based on the Hybrid Index (HI), where M_YY_ have a value of 0 and wild Brook Trout have a value of 1.

##### 2.5.3.1 No Fitness Penalty Scenario (Baseline)

For the baseline scenario, no ID or OD fitness penalties were implemented. Manual suppression began at year 25 through 34 (a range of 10 years) on a cyclical schedule of 3 years on, 2 years off following planned management efforts within Sullivan Creek. A total of 9110 M_YY_ (avg 137 mm fork length) were proportionally released each year from year 28 to 34 (7 years) according to the observed relative abundance of Brook Trout across the treatment reach. Survival and reproduction of M_YY_ were assumed to be equal to their wild counterparts (but see Sections 4.4 and 4.5). Surviving Brook Trout populations were allowed to recover for an additional 32 years (70 years total).

##### 2.5.3.2 Inbreeding Depression (ID) Penalty Scenario

To simulate the impact of ID on the bottleneck magnitude and subsequent recovery of Brook Trout once treatments ceased, a new CDMetaPOP module was developed to implement fitness (measured as mortality) based on observed individual hom_o_ as an indicator of inbreeding depression. The logit relationship between probability of mortality of individuals from hom_o_ was calculated as:

(1) $$\mathrm{ID}\;\text{Probability of Mortality}=\frac{\exp\left(b_{\mathrm{int}}+m\times {\text{hom}}_{o}\right)}{1+\exp\left(b_{\mathrm{int}}+m\times {\text{hom}}_{o}\right)\;}$$

where *b*_int_ is the intercept and *m* is the slope. Using the logit function is standard for modeling survival (or mortality) as it constrains the probability between 0 and 1 (Armstrong and Cassey 2007).

To parameterize the relationship between homozygosity and fitness, we searched for empirical studies estimating the impact of inbreeding on survival in fishes and other taxa. The empirical relationship between inbreeding and fitness remains poorly understood for natural fish populations (Patterson and Pilakouta 2024). There is evidence of inbreeding depression in fish populations (Ryman 1970, Leberg and Firmin 2008, Thrower and Hard 2009, Naish et al. 2013, Rogers et al. 2022) and its magnitude appears to be similar to other taxa (McCune et al. 2004, Thrower and Hard 2009). For example, Thrower and Hard (2009) evaluated Rainbow Trout ( *Oncorhynchus mykiss* ) one generation removed from the wild and found that juvenile to returning adult marine‐phase survival was consistently and significantly lower in inbred individuals, with an estimated number of diploid lethal equivalents (LEs; a lethal equivalent corresponds to the total number of genetic deaths that would result if all the alleles in an individual became homozygous) for anadromous fish of 10.8. McCune et al. (2004) estimated 7.2 diploid LEs for early juvenile survival in wild‐caught and laboratory reared Zebrafish (*Dano rerio*). These values for inbreeding depression are similar to estimates from other vertebrates (Nietlisbach et al. 2019). Thus, inbreeding depression appears to have the potential to influence juvenile survival across vertebrates. We chose to use data from four studies of three wild mammals and a wild bird for which multigenerational estimates of the effect of inbreeding on survival were available, and for which parameter estimates were expressed in the form necessary to satisfy the structure of Equation (1) (Table S2). These included red deer (Huisman et al. 2016; Walling et al. 2011); Soay sheep (Bérénos et al. 2016), black and white ruffed lemurs (Berg 1997, as cited in Kalinowski and Hedrick 1998; Armstrong and Cassey 2007) and North Island robins (Armstrong and Cassey 2007).

Due to the limited number of suitable studies available, and the taxonomic divergence involved, we chose a wide range of values to test sensitivity of model results to strength of ID. We calculated the mean slope across the five studies (mean = 9.57) and then varied the parameter space around the mean by calculating several additional penalty slopes: ±1 SD, ±¼ SD, and ±⅛ SD of the mean slope (Figure 2b). We ran seven simulation scenarios that varied the slope of mortality as a function of the hom_o_ of individual Brook Trout. Slopes ranged from 12.44 for the +1 SD simulations, where 60% homozygosity resulted in a 61% chance of mortality, to 6.71 for the −1 SD simulations, where 60% homozygosity resulted in a 22% chance of mortality. The intercept was calculated as −7.03 to obtain ~50% probability of mortality when ~50% of the genome is homozygous for the highest penalty slope (+1 SD above the mean). All other parameters were held the same as in the baseline scenario.

##### 2.5.3.3 Outbreeding Depression (OD) Penalty Scenario

To simulate the fitness effects of OD due to admixture of M_YY_ alleles on Brook Trout offspring, we used a modified version of the hybrid index (HI; Allendorf et al. 2001). Wild‐type individuals were assigned HI = 1, and M_YY_ HI = 0. Offspring HIs were calculated by averaging those of the parents (e.g., first generation cross of M_YY_ with wild female would have an HI of 0.5; second generation 0.5 HI offspring crossed with wild‐type would have a HI of 0.75). Since we were only interested in OD, we did not incorporate an HI penalty on M_YY_, but only their offspring. The linear correlation between probability of mortality of individuals from OD and HI was calculated as

(2) $$\mathrm{OD}\;\text{Probability of Mortality}=h+\left(p\cdot \mathrm{HI}\right)$$

where *h* is the intercept and *p* is the slope.

We ran three different simulations that varied the slope of the mortality penalty based on HI (Figure 2c). These slopes were based on empirical data from Blouin et al. (2021) that showed 44% lower survival of hatchery steelhead trout offspring compared to wild offspring. Therefore, we set the mortality of individuals with HI = 0 at −0.44 when individuals with HI = 1 are 0 setting up the slope for the higher mortality scenario. The lowest mortality scenario was calculated as half of the highest, with a slope of −0.22 and the medium as an intermediate value between the highest and the lowest slopes, as −0.33. Individuals with an HI of 1 (full wild‐type alleles) had no fitness penalty. All other parameters were held the same as in the baseline.

##### 2.5.3.4 ID + OD Penalties

To simulate the simultaneous impact of ID and OD on Brook Trout recovery, we combined the probability of mortality due to ID with the probability of mortality due to OD as follows:

(3) $$\begin{array}{c} \mathrm{ID}+\mathrm{OD}\;\text{Mortality Probability}=\\ 1-\left[\left(1-\mathrm{ID}\;\text{Probability of Mortality}\right)*\left(1-\mathrm{OD}\;\text{Probability of Mortality}\right)\right] \end{array}$$

We ran eight scenarios that varied the slope of mortality based on both hom_o_ and HI. For this analysis, we selected the slopes from the ID analysis that did not result in population extirpation (±¼ SD of the mean and ±⅛ SD of the mean) and used the minimum and maximum slopes for the OD analysis (Table S3). All other parameters were held the same as in the baseline.

### 2.6 Analysis

To compare the fitness effects of inbreeding, outbreeding, and a combination of both on population bottleneck size and subsequent recovery we calculated 95% confidence intervals (mean ± SD) across replicates for all output metrics; mean *N* over time, the minimum number of individuals observed at any point in the simulation (*MinN*), the percent hom_o_ over time, and the proportion of selection deaths pre‐treatment, during treatments, and at the final year of the simulation, sex ratio, and the percent change in population size (Final Year *N*—Pre‐treatment *N*/Pre‐treatment *N* * 100). Pre‐treatment year was defined as 13, to give the population enough time to stabilize after burn‐in ended at year 10. Bottleneck size was calculated by determining the year for which mean *N* was lowest for each simulation. The final year was 70, except for populations eradicated before that time, for which final year was equal to the year before eradication occurred.

## 3 Results

For the “null scenario” (no treatments, no fitness penalties), all measured metrics remained stable for the full length of the simulation. For all simulations where M_YY_ were introduced, mean *N* was stable until treatments *t* began and then declined toward a population bottleneck until treatments ceased (Figure 3 and Figure S1).

**FIGURE 3:**
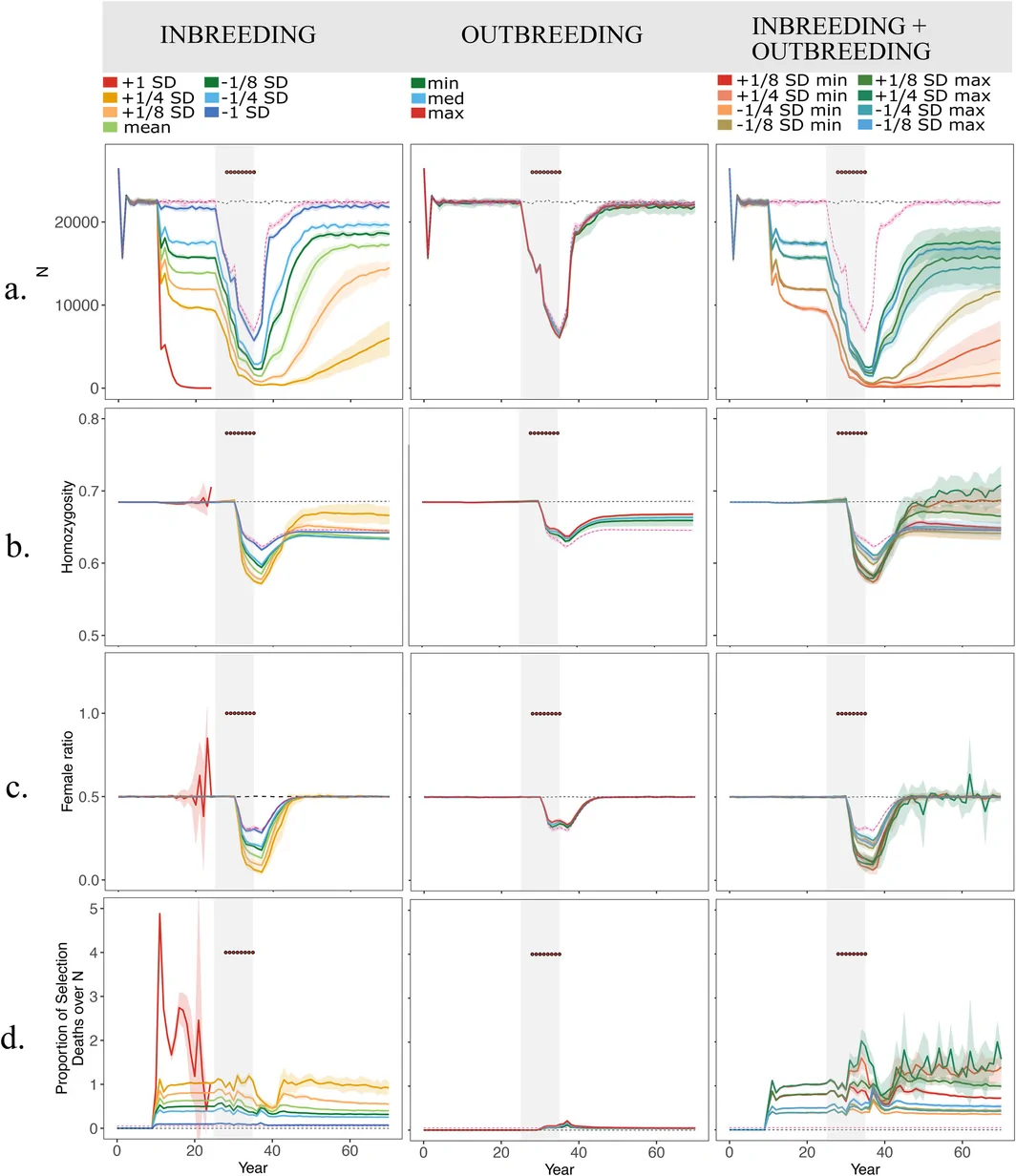
Summary metrics. Baseline and null simulations are shown in dashed magenta and dashed black lines, respectively. Suppression timeframe indicated by vertical grey bar, M_YY_ treatment years shown by red dots. (a) Mean number of individuals (solid lines) and standard deviation (shaded bands). (b) Mean percent hom_o_ (solid lines) and standard deviation (shaded bands) over time based on 5000 loci. (c) Female ratio in the population. (d) Proportion of deaths due to selection mortality relative to all other sources of mortality.

### 3.1 No Fitness Penalty Scenario (“Baseline”)

For the baseline model scenario where there was no fitness penalty, *MinN* was 6921 ± 325 SD (Figure 3). Following treatments *t*, the population recovered by year 48, 10 years after treatments ended. Mean hom_o_ pre‐treatment was 0.684 ± 0.0002 SD, decreased by 8.8% during the bottleneck (mean = 0.632 ± 0.003 SD) and then increased by 4.8%, not returning to pre‐suppression levels (mean = 0.645 ± 0.001). Sex ratio shifted toward male upon M_YY_ introduction but to a lesser extent than the selection scenarios (scenarios 3.2–3.4) and recovered to pre‐treatment levels soon after treatment. Mean percent change in population size resulted in 0.71% ± 1.28 SD higher population at end of the simulation.

### 3.2 Inbreeding Depression Fitness Penalty Scenario (“ID”)

There was a clear effect of ID on the extent of the population bottleneck during treatments. *MinN* varied across slope penalties and increased with decreasing slope penalty. The lowest *MinN* per replicate (without extirpation) occurred in the +¼ SD penalty slope simulation (mean = 356 ± 36 SD) and the highest *MinN* occurred in the −1 SD penalty slope simulation (mean = 5738 ± 364 SD; Figure 3a). The +1 SD penalty slope resulted in rapid extirpation due to the extreme inbreeding effect. Following treatments, the population size recovered to above pre‐treatment levels for all simulations except the lowest and the highest penalty slope simulations (Figure 3a). Due to outbreeding with M_YY_, mean hom_o_ decreased slightly during the population bottleneck and then increased again over time, although significantly lower than pre‐treatment levels for all simulations at the final year of the simulation (Figure 3b). Following introduction of M_YY_, sex ratio shifted toward male in all scenarios, where the smallest and greatest changes (without extirpation) occurred in the −1 SD and +1/4 SD scenario, respectively. Sex ratio recovered to pre‐treatment levels for all scenarios after treatments stopped (Figure 3c). For all simulations except the simulations with the lowest and highest penalty slopes, the proportion of selection deaths over the total population was significantly lower at the final year of simulations compared to pre‐treatment levels (Figure 3d). Percent population change ranged from no recovery for the highest penalty slope to an increase of 15.49% ± 1.61 SD for the mean slope (Table S3).

### 3.3 Outbreeding Depression Fitness Penalty Scenario (“OD”)

*MinN* did not vary among scenarios with different OD fitness penalty slopes during treatments or during population rebound once treatments ended, as the population recovered to just below pre‐treatment levels for all simulations (Figure 3a). Mean hom_o_ decreased slightly during population bottleneck and then increased again; however, not up to pre‐treatment levels for any simulation (Figure 3b). Sex ratio shifted toward male but was not significantly different from the baseline scenario (Figure 3c). The number of selection deaths at the final year of the simulation was higher for all simulations than pre‐treatment because selection is not applied until M_YY_ are introduced (Figure 3d and Figure S2). Mean percent population change ranged from 0.97% ± 1.63 SD lower population size for the medium penalty slope to 2.27% ± 3.17 SD lower population for the minimum penalty slope (Table S3).

### 3.4 ID + OD Combined Fitness Penalties Scenario

For the combined ID and OD penalty simulations, three replicates of the scenario with +¼ SD ID Max OD fitness penalty resulted in eradication after treatments were concluded, with the population failing to recover. The shortest time to eradication among all replicates was 50 years, corresponding to 18 years after treatments ended. *MinN* was lowest in the scenario with the highest penalty ID slope (+¼ SD ID max OD, mean = 124 ± 65 SD; Table S3). No simulations resulted in the population recovery to pre‐treatment levels. Mean hom_o_ decreased slightly during the population bottleneck. Mean hom_o_ pre‐treatment was generally lower (except for +¼ SD max OD—higher, and +¼ SD min OD—same) from mean hom_o_ towards the end of simulations (Figure 3b). Sex ratio shifted toward male the most for +¼ SD min scenario and the least for the −1/8 SD max scenario (Figure 3c). The proportion of selection deaths pre‐treatment did not differ from those after the rebound for any scenario (Figure 3d). Mean percent population change ranged from 97.14% ± 2.10 SD population decrease for the +¼ SD ID Max OD penalty slope simulations to 1.86% ± 10.58 SD decrease for the −¼ SD ID Min OD penalty slope (Table S3).

## 4 Discussion

Using spatially explicit individual‐based simulations, we modeled the demographic and genetic (demogenetic) consequences of incomplete eradication following the introduction of M_YY_ into a non‐native Brook Trout population in a northeast Washington stream. We analyzed the independent and combined effects of inbreeding and outbreeding depression and observed that, when simulated in isolation, inbreeding depression caused higher mortality leading to a more severe decrease in population size and homozygosity, with a more pronounced shift in sex ratio toward male, compared to outbreeding depression. Incorporating the combined effects of inbreeding and outbreeding depression limited population recovery by mitigating the heterosis effect of admixture with genetically divergent M_YY_ in ID‐only scenarios. In these scenarios, the population did not recover to pre‐treatment abundance, demonstrating that the interaction between these mechanisms is relevant for M_YY_ programs. These results suggest that the combination of fitness effects of both inbreeding and outbreeding depression could work in concert to limit the recovery of non‐native populations, once treatments cease, even if they are not completely eradicated.

### 4.1 Inbreeding Depression

The fitness penalty for inbreeding (homozygosity) caused a drastic change in population size, genetic variability, and sex ratio, driving the population through a strong bottleneck. As the fitness penalty increased across scenarios, the population bottleneck increased in severity. In all scenarios, sex ratio eventually returned to 50:50 following treatment. There was also a relationship between the rate of population recovery and the strength of the fitness penalty. In the scenario with the highest penalty, the population was extirpated before the start of the treatments and therefore never recovered, whereas the surviving population with the highest fitness penalty recovered to only approximately half of its original size. Population size in the other scenarios either returned to pre‐treatment levels or exceeded the initial population size in an order proportional to their assigned fitness penalties. The increase in population size after treatment seen in some scenarios is likely due to selection driven by admixture. Admixture with hatchery‐raised M_YY_ can decrease homozygosity by adding new genetic variation to the inbred population, countering the effects of the bottleneck (Bell et al. 2019; McEachern et al. 2011). Selection can act on fitness at different levels, for example against homozygotes at the highest penalty scenario or, as observed in the lowest penalty scenario, with a lower selection mortality during treatments, resulting in a lower negative shift in fitness during the bottleneck.

### 4.2 Outbreeding Depression

The fitness effects due to outbreeding resulted in similar patterns for the three scenarios with outbreeding penalties, although the dip in population size was not as pronounced as in inbreeding. The population bottleneck and the extent of the rebound were lower for the outbreeding simulations compared to the baseline scenario, namely no fitness penalty for maladaptation. This illustrates how non‐native population dynamics, including population size, genetic diversity, and mortality, can be affected by the introduction of M_YY_ through mechanisms that extend beyond the effects of changing the sex‐ratio, such as by introducing harmful genetic variation (e.g., negative heterosis where F1 individuals have lower fitness than parentals and outbreeding having negative fitness influence during recombination events post F1 generations). This leads to a slightly greater bottleneck compared to treatments alone and prevents the population from fully recovering post‐treatment, though recovery was close to pre‐treatment levels (over 90% recovery).

The impact of negative heterosis and outbreeding depression on the survival of hatchery offspring appears to be important, though empirical data are lacking (Edmands 2007; McClelland and Naish 2007). Outbreeding appears to have stronger effects on survival of hatchery hybrids relative to offspring of wild fish (Miller et al. 2004), suggesting the outbreeding fitness penalties we used may have been conservative. However, during a preliminary analysis, we included more extreme fitness demands on our simulations and saw no significant differences for demography, sex ratio, or genetic variation. This suggests there may be a threshold above which an increase in the fitness penalty of outbreeding depression will not result in greater impacts on the bottleneck size and population recovery.

### 4.3 Combination of ID and OD Fitness Penalties

The combination of both inbreeding and outbreeding depression created more variation in the population recovery than either fitness penalty alone to the extent that populations in some replicates failed to recover. Like the ID‐only simulations, the severity of the population bottleneck and the magnitude of the population rebound decreased with the extent of the fitness penalty assigned. However, changes in levels of outbreeding depression did not considerably affect the outcome. Overall, the key insight is that the interaction of inbreeding and outbreeding depression did not allow the population to recover to pre‐treatment levels compared to each fitness penalty in isolation. This suggests that although inbreeding did not affect population dynamics over time, the combination of inbreeding and outbreeding may work to limit non‐native species recovery. Specifically, inbreeding rapidly decreases the overall population size until the bottleneck and outbreeding prevents recovery to pre‐suppression abundance by counteracting the fitness advantage of admixture from genetically divergent M_YY_ (e.g., decreased mean homozygosity), suggesting that outbreeding may be a more important factor to hinder recovery than the magnitude of the bottleneck. In natural systems, evolutionary processes such as inbreeding and outbreeding will rarely act in isolation, therefore their combined and simultaneous effects represent the most likely scenario for suppression and M_YY_ introduction and subsequent non‐native species recovery.

### 4.4 Model Considerations

Since we lacked multigenerational field data on the impacts of inbreeding and outbreeding on survival of Brook Trout, we simulated a wide range of fitness penalties based on empirical data available from mammals and a bird species. While this manuscript was under preparation, Bell et al. (2024) published the first estimates for the relationship between inbreeding and survival in an isolated wild population of salmonids. Their estimates aligned with our approach in showing that inbreeding significantly reduced survival in young males and young‐of‐the‐year, but their estimates did not quantify inbreeding depression in a comparable way.

Although the negative fitness effects of introducing hatchery individuals in the wild have been well studied (Araki et al. 2008), the relative fitness of M_YY_ Brook Trout males in the wild is still not entirely understood, although both equal (Schill et al. 2026) and reduced (Armstrong et al. 2022) fitness of M_YY_ Brook Trout has been reported. For the purposes of this study, we assumed it to be equal to that of wild Brook Trout. Reduced M_YY_ fitness compared to wild Brook Trout has been shown to increase the time to eradication and decrease the likelihood of eradication (Day et al. 2020); therefore, the effects we predicted for the combination of inbreeding and outbreeding depression would likely be weakened by incorporating M_YY_ with lower relative fitness.

When selecting models to use for landscape genetic studies, most available software is restricted either in terms of genomic or spatial complexity. Cumulative effects (e.g., degree of epistasis, demography, behavior, and environment) influence multiple traits and interact to affect the fitness of populations receiving M_YY_ (Edmands 2007; Tallmon et al. 2004). CDMetaPOP is particularly advantageous to predict the effect of treatment strategies because it is possible to calibrate it with empirical data, simultaneously incorporating complex demography, dispersal, genetic data, and spatially heterogeneous landscapes. Future work could include greater genomic complexity into CDMetaPOP to explore the potential fitness disadvantage of M_YY_ on specific traits altered during the initial stages of the artificial manipulation of the sex‐ratio. During this stage of initial M_YY_ Brook Trout introductions, recombination rates can change as the population is purposefully driven through a bottleneck, possibly leading to genetic deterioration and fixation of deleterious mutations (Stelkens and Wedekind 2010). Modeling could help understand how degeneration of the Y chromosome following a bottleneck can spread deleterious mutations into the wild and contribute to eradication of non‐natives facilitated by this “Trojan gene” scenario (Ahrens and Devlin 2011).

### 4.5 Implications for Treatment Strategies

An eradication program simultaneously combining suppression with the introduction of M_YY_ most likely impacts the fitness of the recipient population with interacting evolutionary forces (e.g., drift, migration) that carry implications even if eradication is not ultimately achieved. Although the combined effects of inbreeding and outbreeding depression are difficult to evaluate empirically (Edmands 2007; Tallmon et al. 2004) our modeling found that there may still be a net benefit from both treatments since the population did not fully recover to pre‐treatment levels in the simulation due to reduced fitness of the remnant population.

To reduce uncertainties around the different effects that fitness penalties can have when introducing M_YY_ into a system, it is necessary to determine to what extent M_YY_ fitness differs from wild fish. Evidence to date has produced conflicting findings.

Several studies have reported no significant changes in growth rates, body condition (Unsworth et al. 2022), or survival (Davenport et al. 2024). Others have found evidence of M_YY_ being larger as young of the year, having superior reproductive capacity (Herrera and Cruz 2001; Salirrosas et al. 2017; Wu et al. 2015; Zhou et al. 2023), siring a higher proportion of offspring (Davenport et al. 2024), initiating more mating actions towards females and being more aggressive than M_XY_s (Hamilton et al. 1969; Thresher et al. 2020).

However, other studies have also evidenced lower survival in lab settings (Davenport et al. 2024) and in the wild (Armstrong et al. 2022), and negative impacts on reproductive potential (Kennedy et al. 2018; Kotula‐Balak et al. 2008). Further laboratory studies will be critical to describing fitness effects that are difficult to characterize in wild settings. In applied studies, comparison of empirical outcomes to simulated outcomes (e.g., sex‐ratio, recovery rate) can likewise help to reveal the magnitude of the effects of inbreeding and outbreeding in real systems.

### 4.6 Conclusions

Our results demonstrate how fitness impacts from suppression and M_YY_ implementation may influence overall success of a treatment strategy for eradicating non‐native populations when eradication is not achieved. Ecologically meaningful reductions in population abundance, which are more likely to influence competition with native species, occurred only in scenarios that included both inbreeding and outbreeding effects and only under higher fitness penalties. Under these higher penalty scenarios, full recovery of the surviving population will not occur when eradication is not achieved, indicating that such treatment programs may still show benefit beyond sex ratio manipulation even if they don't achieve full eradication.

## Funding

This work was supported by Seattle City Light. University of Montana's Franke Fresh Water Conservation Initiative. National Science Foundation, 2018112, 1925267. National Institutes of Health, P20GM130418.

## Disclosure

No AI or AI‐assisted tools were used at any stage of this project.

## Ethics Statement

The authors have nothing to report.

## Conflicts of Interest

The authors declare no conflicts of interest.

## Supporting information


**Table S1:** Key parameters and references used in the simulations. Parameters used and/or developed in these simulations with the new CDmetaPOP module are shown in black. The rest of the parameters associated with the brook trout system model are derived from SCL data are already described elsewhere (Day et al. 2021) but provided here in lighter grey text. For additional details see the man body of the manuscript. All input files with complete parameterization can be accessed at https://github.com/ComputationalEcologyLab/YY‐fitness‐Davenport_Alo_et_al. https://github.com/Anonymized/TBD.
**Table S2:** List of available studies with multigenerational estimates of the effect of inbreeding on survival used to calculate the mean slope of −9.57 employed in our study.
**Table S3:** Population sizes mean of 10 runs for each simulation and respective SD of the different bottleneck scenarios and population recovery percentage (± Standard Deviation) for all simulations, including baseline.
**Figure S1:** Population dynamics organized by penalty scenarios at pre‐treatment, bottleneck, and final year for all combinations (error bars = ±SD).
**Figure S2:** Mean proportion of fitness deaths (number of deaths occurred by selection/total population number) pre‐treatment, at the bottleneck, and at the final year of the simulation for all combinations of fitness penalties, including baseline and null (error bars = ±SD). Baseline simulations include treatment but no fitness penalty, null simulations have no treatment and no fitness penalty.

## Data Availability

The data that support the findings of this study are openly available in the repository at https://github.com/ComputationalEcologyLab/YY‐fitness‐Davenport_Alo_et_al.
