# Supplementary material for: Fitness Regulates Non‐Native Species Recovery in a Simulated Trojan MYY Brook Trout Application
Source: Evol Appl. 2026 Oct 14;19(10):e70335. doi: 10.1111/eva.70335 (PMC13662626; doi:10.1111/eva.70335)
Supplement: Supplementary file 1 [file EVA-19-e70335-s001.docx]

Supplemental Data

Supplemental Tables

**Table S1**. Key parameters and references used in the simulations. Parameters used and/or developed in these simulations with the new CDmetaPOP module are shown in black. The rest of the parameters associated with the brook trout system model are derived from SCL data are already described elsewhere (Day et al. 2021) but provided here in lighter grey text. For additional details see the main body of the manuscript. All input files with complete parameterization can be accessed at <https://github.com/ComputationalEcologyLab/YY-fitness-Davenport_Alo_et_al>

| **Input Parameters (Level/Entry)** | **Description** | **Values** | **Reference** |
| --- | --- | --- | --- |
| Run/mcruns | Number of Monte Carlo replication runs per simulation | 10 |  |
| Run/runtime | Total simulation runtime in years | 70 |  |
| Run/cdclimgentime | Start suppression efforts | Starts at year 25 |  |
| Population/cdevolveans (F_Logistic_mean:b_int_) | Inbreeding Fitness Penalty (ID): applies selection to each individual as differential mortality (0-1). Mortality is a function of observed homozygosity (*Hom_O_*) using a logistic function: Differential Mortality = exp (b_int_ + mean * *Hom_O_*)/ 1 + exp (b_int_ + mean * *Hom_O_*). Intercept b_int_ calculated to obtain ~ 50% probability of mortality when 50% of the genome is homozygous for high penalty slope (+1 SD above the mean). Mean slope calculated based on values from empirical data | b_int_ = -7.025  mean = 9.57;  added slopes for  +/- 1 SD of mean,  +/- 1/4 SD of mean  +/- 1/8 SD of mean;  Slopes ranged from 12.44 (+ 1 SD simulations) to 6.71 ( -1 SD simulations) | Huisman et al. 2016, Berenos et al. 2016, Berg (1997, as cited in Kalinowski & Hedrick 1998; Armstrong and Cassey 2007), Walling et al. 2011. |
| Population/cdevolveans (Hindex_Linear_p:h) | Outbreeding Fitness Penalty (OD): Mortality as a function of amount of hatchery YY males admixture per individual (*HI*). Mortality = h + (p * *HI*) where h is the intercept and p is the slope | Hindex_Linear_0.231:0.77 (Min)  Hindex_Linear_0.33:0.67 (Med)  Hindex_Linear_0.429:0.571(Max) | Allendorf et al. 2001 |
| Population /startGenes | Year at when genetic exchange begins | 10 |  |
| Population/(Egg_Mean_ans, Egg_Mean_par1, Egg_Mean_par2) | Fecundity measured as number of eggs per female modeled as a function of size using a linear model with intercept (Egg_Mean_par1) and slope (Egg_Mean_par2) to estimate mean egg number | Number of eggs = 3.78 * Length (mm) -455 | SCL data; modeled after Downs et al. 1997 |
| Population/(  mature_defaultAge, mature_eqn_slope, mature_eqn_int) | These are the slope and intercept parameters that describe the probability of maturation as a function of size. P(mature) = exp(mature_eqn_int + mature_eqn_slope * Length) / (1 + exp(mature_eqn_int + mature_eqn_slope * Length))  For YY males, they are introduced forced to mature at age 1 | Wild: P(mature) = exp ((-6.313 + 0.0539 * Length) / (1 +exp ((-6.313 + 0.0539 * Length)); YY males: Forced to mature at age 1, 1 year following release | SCL data; modeled after Downs et al., 1997 |
| Population/(growth_option, growth_Loo, growth_R0) | ‘temperature’ - the von Bertalanffy function is modified by parameters that are fit to temperature as well. | Newsize (mm) = 400 * (1 – exp( -0.47 * (age + 1-0.075))); maxtemp = 10.5; tempcv = 0.33 (see CDMetaPOP user’s manual) | Landguth et al., 2017; von Bertalanffy, 1938; Seattle City Light |
| Patch/X,Y | Number of patches and location | 152 patches divided into ~200 m reaches (Sullivan Creek only) | Day et al. 2020 |
| Patch/K | Carrying capacity | Variable: based on multi-pass depletion surveys from 2013 – 2017 and set so that N from the simulation model = estimated abundance. | Walston 2018 |
| Patch/MortalityBack | Electrofishing mortality | Based on capture probability of 0.85 |  |
| Patch/N0 | Defines number of YY male introduction numbers each year (from year 28 for 7 yrs) | Total of 9,110 age-0 distributed across patches with carrying capacity defined by SCL empirical data. | Day et al. 2020 |
| Patch/MortBack | Mortality starts at year 25 as indicated from Run/cdclimgentime, and here it defines the years at which it is applied | Mortality lasts for 13 yrs total with a 3yrs on/2yrs off schedule. |  |
| Patch/Allele frequency files/Frequency (at first locus) | This is the hybrid index or allele frequency for each individual. It is the average of the parent’s hybrid index where at initialization users need to specify the initial ‘HIndex’ values for each allele frequency file used using the first locus AA = 1.0 & aa = 0.0. Hindex values of offspring are calculated as average of parents. | Wild: H-index=1  YY male: H-index=0 | Allendorf et al. 2001 |
| Patch/Allele frequency files/Frequencies | Wild: Originally 240 loci generated from genotypes from seven Sullivan Creek populations file; YY male: Originally 219 loci generated from genotypes from Hayspur Hatchery. | 5000 replicated loci from Wild and YY male sequenced loci | Davenport K, 2023 |
| Class/(Body Size Mean (mm), Body Size Std (mm)) | Mean length and standard deviation (mm) of each age class at initialization | Wild: 60, 110, 140, 180, 200, 230, 240, 250**;** YY male**:** All 137 | SCL data |
| Class/Age Mortality Out % | Class specific mortality; as an age check on old fish, 50% mortality applied on all individuals age 7+ | 50 | SCL data |
| Population/Distance matrix files (mate_cdmat, migrateout_cdmat, migrateback_cdmat, stray_cdmat, disperseLocal_cdmat) | Migration, mating, and straying movements. Distance moved each year, based on Pareto distribution. Resistance to movement surfaces. Isolation-by-distance + physical barriers (i.e., dams, culverts). Max mating movement = 1 km. Maximum straying and migration distance is determined by Pareto distribution (i.e., very rare long-distance movements) | Distance matrix based on Pareto distribution distances | Landguth et al., 2012; Landguth, 2010; Letcher et al., 2015 |

**Table S2.** List of available studies with multigenerational estimates of the effect of inbreeding on survival used to calculate the mean slope of -9.57 employed in our study.

| **Species** | **Component of Survival** | **Slope** | **Citation** |
| --- | --- | --- | --- |
| deer | survival age 0-2 | -13.5 | Huisman et al. 2016 |
| sheep | first year survival | -5.55 | Berenos et al. 2016 |
| sheep | annual survival | -10.75 | Berenos et al. 2016 |
| sheep | first year survival | -9.36 | Berenos et al. 2016 |
| sheep | annual survival | -11.7 | Berenos et al. 2016 |
| deer | winter first year survival | -9.74 | Walling et al., 2011) |
| black and white ruffed lemurs | survival | -10.713 | Berg (1997, as cited in Kalinowski & Hedrick 1998; Armstrong and Cassey 2007) |
| North Island Robins | juvenile survival | -5.28 | Armstrong and Cassey 2007 |
| Mean |  | -9.57 |  |

**Table S3**. Population sizes mean of 10 runs for each simulation and respective SD of the different bottleneck scenarios and population recovery percentage (+/- Standard Deviation) for all simulations, including baseline.

| Scenario | Simulation | Bottleneck Year | Bottleneck N | bottleneck sd | Population recovery (+/- SD) (%) |
| --- | --- | --- | --- | --- | --- |
| Suppression + Treatment | Baseline | 35 | 6921 | 325 | 0.71 (1.28) |
| +1 SD | ID | 24 | 2 | NA | NA |
| +1/4 SD | ID | 37 | 356 | 36 | -46.55 (18.31) |
| +1/8 SD | ID | 37 | 770 | 113 | 10.29 (6.24) |
| mean | ID | 37 | 1462 | 133 | 15.49 (1.61) |
| -1/8 SD | ID | 36 | 2269 | 141 | 12.30 (1.55) |
| -1/4 SD | ID | 36 | 2874 | 175 | 9.94 (0.96) |
| -1 SD | ID | 35 | 5738 | 364 | -0.08 (1.04) |
| min | OD | 35 | 6421 | 551 | -2.27 (3.17) |
| med | OD | 35 | 6418 | 337 | -0.97 (1.63) |
| max | OD | 35 | 6091 | 286 | -1.91 (1.87) |
| +1/8 SD min | ID + OD | 37 | 580 | 86 | -12.32 (7.72) |
| +1/4 SD min | ID + OD | 37 | 236 | 38 | -84.05 (15.58) |
| -1/4 SD min | ID + OD | 36 | 2483 | 126 | -1.86 (10.58) |
| -1/8 SD min | ID + OD | 36 | 1819 | 251 | -5.30 (19.00) |
| +1/8 SD max | ID + OD | 37 | 414 | 72 | -55.42 (17.62) |
| +1/4 SD max | ID + OD | 43 | 124 | 65 | -97.14 (2.10) |
| -1/4 SD max | ID + OD | 36 | 2096 | 187 | -6.53 (1.42) |
| -1/8 SD max | ID + OD | 37 | 1455 | 173 | -11.92 (16.17) |

Supplemental Figures

Figure S1. Population dynamics organized by penalty scenarios at pre-treatment, bottleneck, and final year for all combinations (error bars = ± SD).

Figure S2. Mean proportion of fitness deaths (number of deaths occurred by selection/total population number) pre-treatment, at the bottleneck, and at the final year of the simulation for all combinations of fitness penalties, including baseline and null (error bars = ± SD). Baseline simulations include treatment but no fitness penalty, null simulations have no treatment and no fitness penalty.

**References**

Allendorf, F., Leary, R., Spruell, P., Wenburg, J., 2001. The problems with hybrids: setting conservation guidelines. Trends Ecol. Evol. 16, 105. https://doi.org/10.1016/0305-4403(79)90038-4

Armstrong, D.P., Cassey, P., 2007. Estimating the effect of inbreeding on survival. Anim. Conserv. 10, 487–492. https://doi.org/10.1111/j.1469-1795.2007.00139.x

Bérénos, C., Ellis, P.A., Pilkington, J.G., Pemberton, J.M., 2016. Genomic analysis reveals depression due to both individual and maternal inbreeding in a free-living mammal population. Mol. Ecol. 25, 3152–3168. <https://doi.org/10.1111/mec.13681>

Davenport, Kaeli Alexis. *A Multidisciplinary Approach to the Management of a Non-Native Trout Species*. University of Montana, 2023.

Day, C.C., Landguth, E.L., Simmons, R.K., Baker, W.P., Whiteley, A.R., Lukacs, P.M., Bearlin, A., 2020. Simulating effects of fitness and dispersal on the use of Trojan sex chromosomes for the management of invasive species. J. Appl. Ecol. 57, 1413–1425. <https://doi.org/10.1111/1365-2664.13616>

Downs, C.C., White, R.G., Shepard, B.B., 1997. Age at sexual maturity, sex ratio, fecundity, and longevity of isolated headwater populations of westslope cutthroat trout. North Am. J. Fish. Manag. 17, 85–92.

Huisman, J., Kruuk, L.E.B., Ellisa, P.A., Clutton-Brock, T., Pemberton, J.M., 2016. Inbreeding depression across the lifespan in a wild mammal population. Proc. Natl. Acad. Sci. U. S. A. 113, 3585–3590. <https://doi.org/10.1073/pnas.1518046113>

Kalinowski, S.T., Hedrick, P.W., 2001. Inbreeding depression in captive bighorn sheep. Anim. Conserv. 4, 319–324. <https://doi.org/10.1017/S1367943001001378>

Landguth, E., Hand, B., Glassy, J., Cushman, S., Sawaya, M., 2012. UNICOR: a species connectivity and corridor network simulator. Ecography (Cop.). 35, 9–14.

Landguth, E.L., 2010. CDPOP: a spatially explicit cost distance population genetics program. Mol. Ecol. Resour. 10, 156–161.

Landguth, E.L., Bearlin, A., Day, C.C., Dunham, J., 2017. CDMetaPOP: an individual-based, eco-evolutionary model for spatially explicit simulation of landscape demogenetics. Methods Ecol. Evol. 8, 4–11. https://doi.org/10.1111/2041-210X.12608

Letcher, B.H., Schueller, P., Bassar, R.D., Nislow, K.H., Coombs, J.A., Sakrejda, K., Morrissey, M., Sigourney, D.B., Whiteley, A.R., O’Donnell, M.J., 2015. Robust estimates of environmental effects on population vital rates: an integrated capture–recapture model of seasonal brook trout growth, survival and movement in a stream network. J. Anim. Ecol. 84, 337–352.

von Bertalanffy, L., 1938. A quantitative theory of organic growth (inquiries on growth laws, II). Hum. Biol. 10, 181–213.

Walling, C.A., Nussey, D.H., Morris, A., Clutton-Brock, T.H., Kruuk, L.E.B., Pemberton, J.M., 2011. Inbreeding depression in red deer calves. BMC Evol. Biol. 11. https://doi.org/10.1186/1471-2148-11-318

Walston, J. 2018. Sullivan Creek non-native fish suppression project, 2017 report, Boundary Hydroelectric Project (FERC No. 2144). Kalispel Tribe of Indians, Usk, WA, USA.
